# ADAPTING TO THE HEALTH RESEARCH MENTORING LANDSCAPE: THE ENHANCING RESEARCH FOR ONLINE LEARNERS (ENROLL) PROGRAM

**DOI:** 10.1093/geroni/igad104.0938

**Published:** 2023-12-21

**Authors:** Matthew Peterson

**Affiliations:** University of North Carolina Wilmington, Wilmington, North Carolina, United States

## Abstract

Engaging students in mentored research has contemporary challenges, including virtual environments and student disengagement. This provides an opportunity to develop novel methods to attract and support students in health research, with the goal of encouraging a career in the health sciences. The overarching objective of EnROLL is to attract, support, and engage on-line and on-campus learners in faculty-led, interdisciplinary health science research. The specific aims of EnROLL are, 1) to develop a focused program and curriculum in faculty-led research to engage online graduate and underserved undergraduate students in health research, 2) to develop and implement an effective model of research mentor training for dissemination, and 3) to assess the value in the program as a model to disseminate for engaging students in faculty-led health research. Now in the second year of funding, attracting students into the mentoring structure has been the biggest challenge. To address this challenge, novel strategies for student recruitment and engagement have been implemented. A stipend-supported scholars program and novel intergenerational events are two examples. Engaging external institutional partners has also yielded differing perspectives and collaborations. This program’s recruitment challenges have yielded new programmatic strategies and unforeseen opportunities. The need for creative and adaptable programming will be highlighted and discussed.

